# 

**Published:** 1893-07

**Authors:** 


### CONTENTS.

CLINICAL LECTURE—
Surgical Clinic at Harlem Hospital. By Thomas H. Manley, M. D., New York.. 257
ORIGINAL COMMUNICATIONS—
State and Municipal Hygiene: With a Plea for a State Board of Health. By Jas. C. Avary, M. D., Atlanta, Ga........ 261
The Relations of Operative Gynecology to Insanity. By A. H. McFarland, M.D., Jacksonville, Ill........ 269
Four Women who Refused Oophorectomy, and their Subsequent Histories. By H. McHatton, M. D., Macon, Ga........ 273
SOCIETY REPORTS—
Philadelphia Academy of Surgery........ 276
CORRESPONDENCE—
New York Letter........ 282
Valvular Insufficiency and Anasarca........ 287
An Obstinate Case of Constipation........ 288
EDITORIAL—
Meeting of the American Medical Association........ 290
Sir Joseph Lister on Antisepsis........ 291
SELECTIONS AND ABSTRACTS—
Skin Diseases and Syphilis........ 294
Surgery........ 297
General Medicine and Therapeutics........ 302
Obstetrics and Gynecology........ 306
Eye, Ear, Nose and Throat........ 310
MEDICAL ITEMS........ 313
BOOK REVIEWS........ 316
INDEX TO ADVERTISEMENTS........ xii
ITEMS OF INTEREST........ xi
PREMIUM OFFERS........ xv

